# Early goal-directed resuscitation for patients with severe sepsis and septic shock: a meta-analysis and trial sequential analysis

**DOI:** 10.1186/s13049-016-0214-7

**Published:** 2016-03-05

**Authors:** Li-bing Jiang, Mao Zhang, Shou-yin Jiang, Yue-feng MA

**Affiliations:** Emergency Medicine Research Institute of Zhejiang University; Emergency Medicine Center, Second Hospital Affiliated to Medical College, Zhejiang University, 88# Jie Fang road, Shang Cheng district, 310009 Hangzhou, China

## Abstract

**Background:**

The aim of this study was to explore whether early goal-directed therapy (EGDT) was associated with a lower mortality rate in comparison to usual care in patients with severe sepsis and septic shock.

**Methods:**

PubMed, EMBASE, Cochrane library and a Chinese database (SinoMed) were searched systematically to identify randomized controlled trials (RCTs) comparing standard EGDT with usual care in resuscitation of patients with severe sepsis and septic shock and the search time could date back to the publication of the study by Rivers in 2001. The study selection, data extraction and methodological evaluation were performed by two investigators independently. The primary outcome was all-cause mortality. The present meta-analysis had been registered in PROSPERO (CRD42015017667).

**Results:**

Our meta-analysis identified 6 studies and enrolling 4336 patients. There was no significant difference in mortality between the two groups, and the pooled odds ratio (OR) was 0.83 (95 % confident interval, CI, 0.64–1.08) with significant heterogeneity (*p* = 0.02, I^2^ = 64 %). However, the pooled OR of 3 multicenter RCTs was 1.03 (95 % CI, 0.89–1.21) with no heterogeneity (*p* = 0.78, I^2^ = 0 %). The effects of EGDT on length of stay in the emergency department and intensive care unit were uncertain, and there was no effect of EGDT on hospital length of stay. There were no differences of mechanical ventilation rate and renal replacement therapy rate between the two groups, and patients in the EGDT group were more admitted in ICU than patients in the control group. During the early 6-h intervention period, patients in the EGDT group received more intravenous fluids, had a higher vasopressor usage rate, higher dobutamine usage rate and higher blood transfusion rate, than patients in the control group. Finally, there was no difference in the incidence of adverse events between the two groups, and the pooled OR was 1.06 (95%CI 0.80–1.39) with moderate heterogeneity (I^2^ = 62 %, *p* = 0.07).

**Discussion:**

Our meta-analysis showed that the application of EGDT was not associated with lower mortality rate currently. However it does not mean that it is useless of EGDT in patients with sever sepsis and septic shock. On the contrary, there was no difference in mortality rate between the two groups may be due to the improvement of therapeutic strategies in these patients. And the results may be related to the different compliance rate of EGDT resuscitation bundle.

**Conclusions:**

The current evidence does not support the significant advantage of Early goal-directed therapy (EGDT) in the resuscitation of patients with severe sepsis and septic shock.

**Electronic supplementary material:**

The online version of this article (doi:10.1186/s13049-016-0214-7) contains supplementary material, which is available to authorized users.

## Background

Sepsis, especially severe sepsis and septic shock are associated with a high rate of death and complications. It has been reported that the annual incidence of severe sepsis and septic shock in the United States is up to 300 cases per 100,000 people [1–3]. Despite the advances in the therapy of severe sepsis and septic shock, the mortality of these patients remains high, ranging from 20 % to 50 % [4–6]. In 2001, a randomized, non-blinded, controlled trial conducted by Rivers reported that patients with severe sepsis and septic shock presenting to the emergency department had a lower mortality rate, if they received a specific 6 h resuscitation bundle of early-goal directed therapy (EGDT), and the absolute risk reduction was 16 % [7]. Therefore, EGDT was recommended by the 2004, 2008 and 2012 surviving sepsis campaigning (SSC) guidelines [8–10]. Meanwhile, several observational studies also found the survival benefit of standard EGDT or modified EGDT in comparison to usual care [11–29]. However, the effect of EGDT was questioned in three multicenter randomized controlled trials, namely ProCESS, ARISE and ProMISe. The authors reported that EGDT did not reduce the mortality rate of patients with severe sepsis and septic shock as compared with usual care [6, 30, 31]. In a meta-analysis, Gu reported that EGDT significantly reduces the overall mortality of patients with sepsis. However, several limitations of this study should be noted: firstly, the newest studies of ARISE and ProMISe study were not included; secondly, the included studies in this meta-analysis were published between 1992 and 2014, during which time the standard care of sepsis as well as the definition of sepsis had changed a lot; Thirdly, this meta-analysis ignored the fact that the pooled results of China-centric studies were significantly different from the pooled results of America and Australia-centric studies; Finally, several studies included in this meta-analysis did not meet the inclusion criteria (one was not a RCT but a before-and-after study; one included patients with systemic inflammatory response syndrome not sepsis and one evaluated modified EGDT not standard EGDT) [32]. The objective of our study was to systematically evaluate the effect of EGDT on patients with severe sepsis and septic shock.

## Methods

We had registered the present meta-analysis protocol in PROSPERO (http://www.crd.york.ac.uk/PROSPERO/; CRD42015017667). Our meta-analysis was conducted and reported based on The Preferred Reporting Items for Systematic Reviews and Meta-Analyses (PRISMA) statement [33].

### Data sources

The following databases: PubMed, EMBASE, the Cochrane library, and a Chinese database (SinoMed) were searched systematically to identify eligible studies, which were published since the publication of the study by Rivers in 2001 [7]. The search process of PubMed was showed in Additional file 1. Additionally, the bibliography of each relevant study was also examined to limit the potential publication bias. The last update search on 5 April 2015 and there was no language restriction.

### Study selection and data extraction

**Inclusion criteria (P**atient, **I**ntervention, **C**omparison, **O**utcome, **S**tudy design**)**

### Patients

Patients with severe sepsis or septic shock were included. And the diagnosis of severe sepsis and septic shock was made according to appropriate guidelines [34, 35].

### Interventions

#### Standard EGDT

The standard EGDT was defined in the SSC guideline including the central venous pressure (CVP) should achieve 8 to 12 mmHg, mean arterial pressure (MAP) should achieve 65 to 90 mmHg, urine output should achieve 0.5 ml/kg/h or more, and central venous oxygen saturation (ScvO_2_) should achieve 70 % or above within the first 6 h of interventions [8].

### Comparison

#### Usual care

The usual care was defined as conventional treatments which were at the discretion of the clinicians.

### Outcomes

The primary endpoint was all-cause mortality. If several mortality rates were reported in one study, we preferred to use the primary one. The secondary endpoints included: the length of stay in emergency department (ED), intensive care unit (ICU) and hospital; mechanical ventilation rate, renal replacement therapy rate and ICU admission rate; the intravenous fluids volume, vasopressor use rate, dobutamine use rate, blood transfusion rate in the first 6 h, between 6 and 72 h and between 0 and 72 h; the incidence of adverse events.

### Study design

#### Randomized controlled trials (RCTs)

The complete processes of study selection and data extraction were conducted by two investigators independently. The study selection was performed in three phases. Firstly, two investigators independently screened the titles and abstracts of all search results, then removed apparently irrelevant studies, and imported those potentially eligible studies into the Endnote; Secondly, the duplicated studies were excluded; Thirdly, the remaining articles were read in full text. Meanwhile, the following data were extracted from each eligible study using a pre-defined data extraction sheet: the first author, year of publication, characteristics of patients in the intervention and control group, characteristics of resuscitation strategies in the intervention and control group, mortality rate at different follow-up time, other secondary outcome endpoints and items for quality evaluation. Any discrepancy was resolved by discussion with a third investigator.

### Quality assessment

The methodological qualities of all included studies were assessed by two investigators independently using the Cochrane risk of bias tool. All eligible studies were judged to be “high”, “unclear”, or “low” risk of bias based on the following domains: random sequence generation; allocation concealment; blinding of patients and personnel; incomplete outcome data; selective outcome reporting; other bias. Any one or more of the above domains were considered to be at high risk of bias, and then the study would be judged to be at high risk of bias [36, 37]. Only when all key domains were considered to be at low risk of bias, would be the study judged to be at low risk of bias. In other cases, the risks of bias were judged to be unclear [36, 37].

### Data analysis

The primary endpoint was all-cause mortality. We prefer to use all-cause mortality which was the primary endpoint in individual study. The metrics of odds ratio (OR) and weighted mean difference (WMD) were used to analysis dichotomous variables and continuous variables separately. Heterogeneity between studies was assessed qualitatively using the Mantel-Haenszel chi-square test and quantitatively using the I ^2^ statistics [38, 39]. When P ≤ 0.1 or I^2^ ≥ 50 %, there was significant heterogeneity between included studies, and random-effect model was used, otherwise the fixed-effect model was used [40]. The publication bias was evaluated using funnel plot and Egger’s test [41].

### Post hoc subgroup analysis

ProCESS, ARISE and ProMISe are published recently and are consistent in study design [6, 30, 31]. Thus, we pooled the primary and secondary endpoints repeatedly based on the above three studies, when required data were available.

In actually, the results of a meta-analysis are the accumulation of results of all eligible studies. Therefore, it is vulnerable to random errors deriving from sparse data and repetitive testing of accumulated data [42]. In addition, enough sample size is necessary for a meta-analysis. Trial sequential analysis (TSA) is the best available approach to calculate the optimal required information size (meta-analysis sample size) for a meta-analysis [43]. TSA was performed in our meta-analysis based on the average baseline mortality rate of control group, a relative risk reduction of 20 [6, 30, 31], 80 of power, and a type I error of 5 %. The random errors adjusted monitoring boundaries were constructed to determine whether new clinical trials were still required to confirm the present conclusion [43–44]. Statistical analyses were performed on Revman 5.2.5, STATA (SERIAL NO. 40120519635) and TSA V.0.9 β (http://www.ctu.dk/tsa/).

## Results

**Fig. 1:**
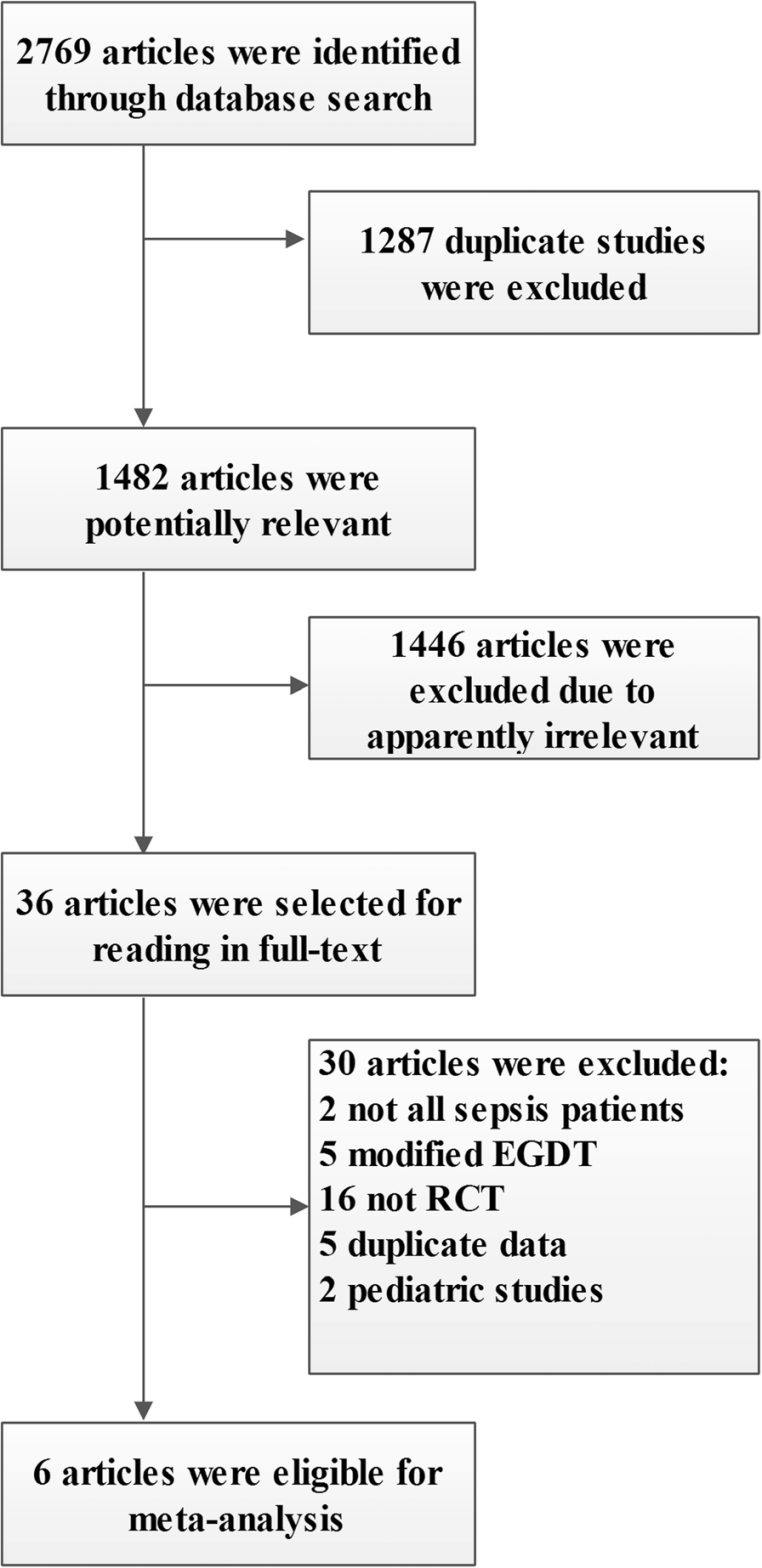
Flow chart for study selection

After excluding the reduplicative, non-relevant studies and other studies that did not meet our inclusion criteria, 6 studies comparing standard EGDT with usual care in patients with severe sepsis and septic shock were eligible [6, 7, 30, 31, 45, 46]. The flow chart of literature selection and corresponding exclusion reasons are summarized in Fig. 1. Among the included studies, 2 studies from China [45, 46], 2 studies from the USA [6, 7], 1 study from Australia [30], and 1 study from the UK [31]. A total of 2160 patients in the EGDT group and 2176 patients in the control group were included. Two studies included patients with severe sepsis and septic shock who stayed in the intensive care units (ICU) [45, 46], and the remaining four studies included patients with severe sepsis and septic shock who presenting to the emergency department (ED) [6, 7, 30, 31]. All studies started EGDT for resuscitation within 6 h. The detailed characteristics of individual eligible study are listed in Table 1.

**Table 1:** Characteristics of included studies in the meta-analysis

Study/Year	Country	Setting	No. of patients		Resuscitation goals		Outcome^a^
			Intervention	Control	Intervention	Control	
Rivers [7]/2001	America	Eemergency department	130	133	SvO_2_ ≥ 70 % CVP 8–12 mmHg MAP 65–90 mmHg Urine volume ≥0.5 ml/kg/h	CVP 8–12 mmHg MAP 65–90 mmHg Urine volume ≥0.5 ml/kg/h	Hospital mortality
Wang [45]/2006	China	ICU	16	17	SvO_2_ ≥ 70 % CVP 8–12 mmHg MAP ≥65 mmHg Urine volume ≥0.5 ml/kg/h	MAP ≥65 mmHg Urine volume ≥0.5 ml/kg/h	14 days mortality
Yan [46]/2010	China	ICU	157	146	ScvO_2_ ≥ 70 % CVP 8–12 mmHg SBP >90 mmHg MAP ≥65 mmHg Urine volume ≥0.5 ml/kg/h	CVP 8–12 mmHg SBP >90 mmHg MAP ≥65 mmHg Urine volume ≥0.5 ml/kg/h	ICU mortality
ProCESS [6]/2014	America	Eemergency department	439	456	ScvO_2_ ≥ 70 % CVP 8–12 mmHg MAP 65–90 mmHg Urine volume ≥0.5 ml/kg/h	Usual care	Hospital mortality
ARISE [30]/2014	Australia/New Zealand	Eemergency department	793	798	SpO_2_ ≥ 93 % ScvO_2_ ≥ 70 % CVP (Self-ventilation) >8 CVP (Non-invasive/invasive MV) >12 mmHg MAP 65–90 mmHg Urine volume ≥0.6 ml/kg/h Haematocrit ≥30 %	Usual care	90 days mortality
ProMISe [31]/2015	UK	Eemergency department	625	626	ScvO_2_ ≥ 70 % CVP 8–12 mmHg MAP 65–90 mmHg Urine volume ≥0.5 ml/kg/h	Usual care	90 days mortality

*CVP* central venous pressure, *MAP* mean arterial pressure, *ScvO*_*2*_ central venous oxygen saturation

^a^primary outcome

### Methodological quality

**Fig. 2:**
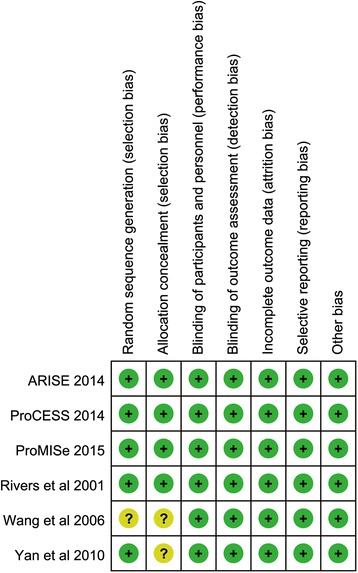
Risk of bias summary

Four included studies were rated as low risk of bias [6, 7, 30, 31] and the remaining studies were rated as unclear risk of bias due to the insufficiency of definitive describe of randomization and allocation concealment [45, 46]. The methodological qualities of all included studies are presented in Fig. 2.

### Primary outcome: mortality

**Fig. 3:**
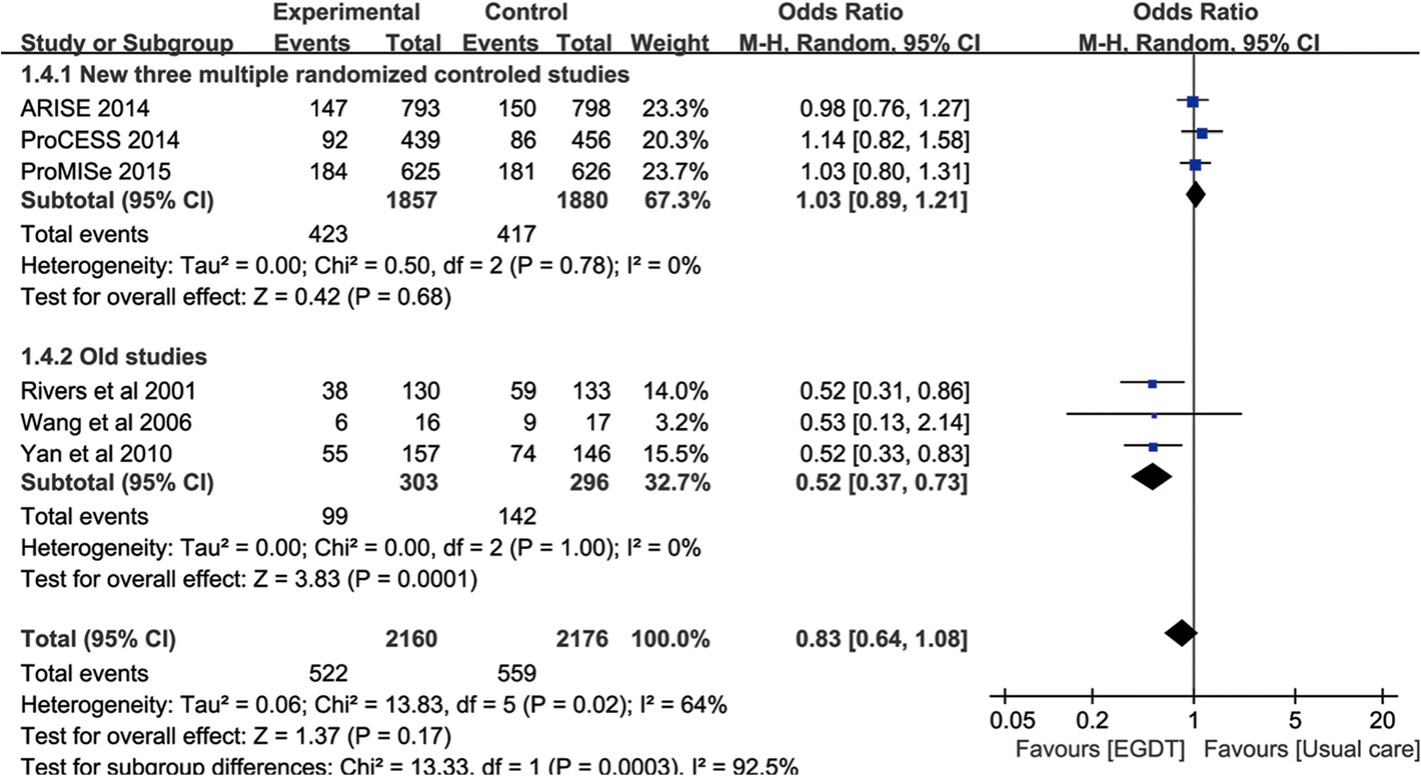
Forest plot showing the effects of early goal-direced therapy on all-cause mortality in patients with severe sepsis and septic shock

The mortality of patients in the standard EGDT group was 24.2 (522/2160) and the mortality rate of patients in the usual care group was 25.7 % (559/2176) [6, 7, 30, 31, 45, 46]. The pooled results indicated that, there was no significant difference in overall mortality rate between study group and control group, and the pooled odds ratio was 0.83 (95 % confident interval, CI, 0.64–1.08) in the random-effect model, and 0.91 (95 % CI, 0.79–1.05) in the fixed effect model. However, there was significant heterogeneity between studies (*p* = 0.02, I^2^ = 64 %) (Fig. 3). What should be noted here was that the average mortality rate of the ProCESS, ARISE, and ProMISe studies [6, 30, 31] was significantly lower than other old studies (22.2 % vs 48 %) [7, 45, 46]. In a post hoc subgroup analysis of these three RCTs [6, 30, 31], the efficacy of EGDT compared with usual care was 1.03 (95 % CI, 0.89–1.21) in the fixed effect model, with no heterogeneity (*p* = 0.78, *I*^*2*^ = 0 %) (Fig. 3). On the contrary, the pooled OR of the remaining old studies was 0.52 (95 % CI, 0.37–0.73) in the fixed effect model, with no heterogeneity (*p* = 1.0, *I*^*2*^ = 0 %) (Fig. 3).

**Fig. 4:**
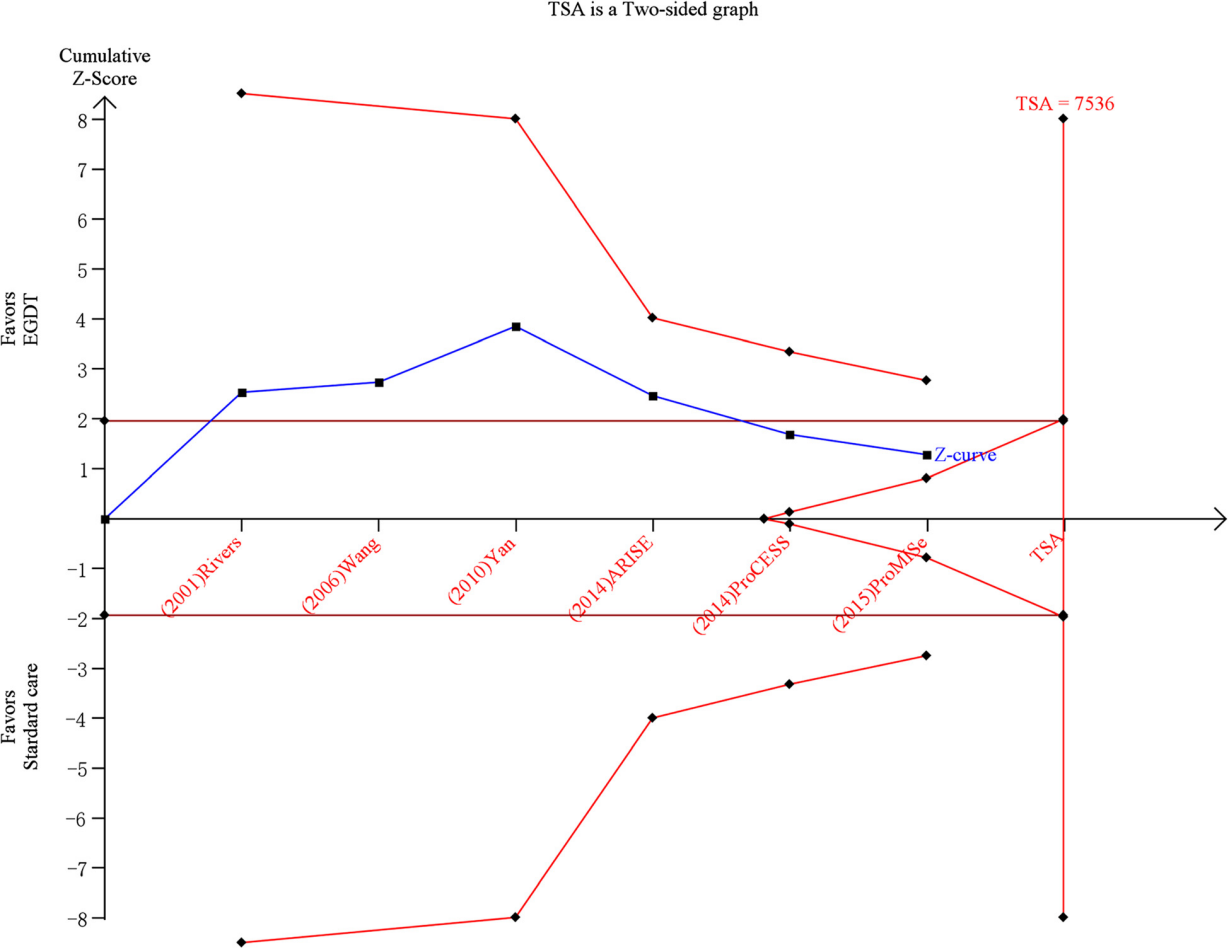
Trial sequential analysis of all-cause mortality in patients with severe sepsis and septic shock. Trial sequential analyses assessing the effect of early goal-direced therapy on all-cause mortality in 6 studies. The diversity-adjusted required information size was based on a relative risk reduction of 20; an alpha of 5; a beta of 2 and an event proportion of 25.7 % in the control arm. The blue cumulative z curve was constructed using a random effects model

**Fig. 5:**
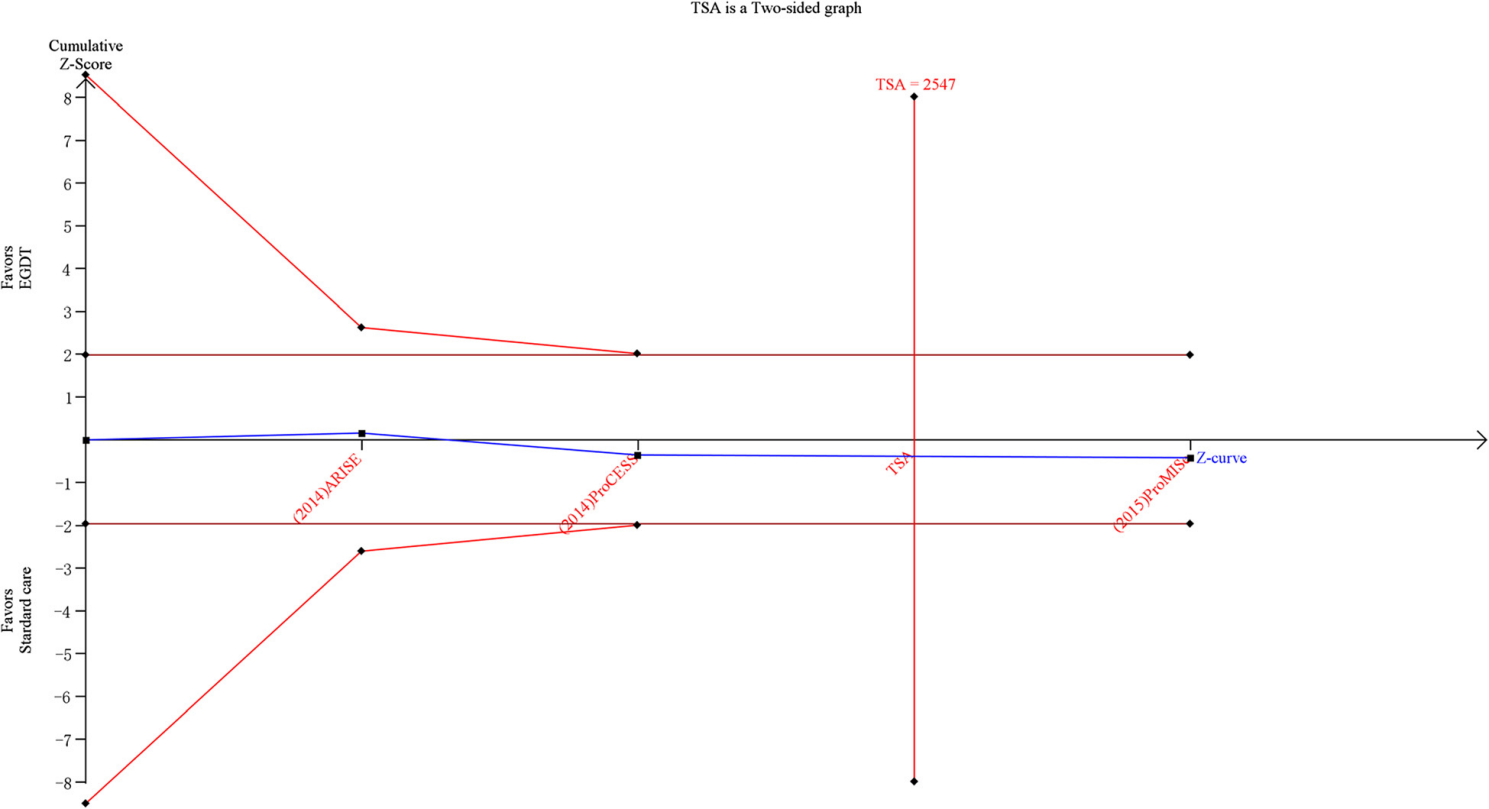
Trial sequential analysis of all-cause mortality in patients with severe sepsis and septic shock of ProCESS, ARISE and ProMISe. Trial sequential analyses assessing the effect of early goal-direced therapy on all-cause mortality in 3 multicenter harmonious studies. The diversity-adjusted required information size was based on a relative risk reduction of 20; an alpha of 5; a beta of 2 and an event proportion of 22.2 %in the control arm. The blue cumulative z curve was constructed using a random effects model

TSA was performed for all 6 studies and 3 homogeneous RCTs (ProCESS, ARISE, and ProMISe). In the former analysis, TSA showed that the diversity adjusted information size was 7536 and the cumulative Z-curve both did not surpassed the conventional boundary and trial sequential monitoring boundary for benefit or harm, as well as did not achieve the optimal information size which indicated the current results were not robust, and further clinical trials were required. However, the above results of TSA were gained based on 6 studies with significant heterogeneity (Fig. 4). The TSA of 3 homogeneous RCTs showed the diversity adjusted information size was 2457 and the cumulative Z-curve did not surpassed the conventional boundary and the trial sequential monitoring boundary for benefit or harm, but achieved the optimal information size which indicated the current results were robust, namely there was no significant difference in mortality between the two groups (Fig. 5).

Additionally, as mortality rates at different follow-up time-points were reported in each included study, we pooled these results separately and found there was also no significant difference in ICU-mortality (OR 0.68 95 CI, 0.44–1.06), hospital mortality (OR 0.85 95 CI, 0.61–1.17), 28-day mortality (OR 0.72, 95 CI, 0.51–1.04), 60 mortality (OR 0.82, 95 CI, 0.41–1.64) and 90-day mortality (OR 0.98, 95 % CI, 0.84–1.14) (see Additional file 2) between two groups.

### Secondary endpoint-1: length of stay in the ED, ICU, and hospital

The ED-LOS, ICU-LOS and hospital-LOS were reported as median and interquartile range in some studies, which might limit the quantitatively pool of results (Table 2). ED-LOS was reported in the study by Yan [46] and the ARISE and ProMISe studies [30, 31]. In the ARISE study, the authors reported that EGDT was associated with shorter ED time, however, this effect of EGDT was not found in Yan’s study and the ProMISe study [30]. ICU-LOS was reported in the ProCESS, ARISE and ProMISe studies [6, 30, 31]. And the pooled results showed that there was no effect of EGDT on ICU-LOS (MD 0.05, 95%CI −0.43 - 0.53) with no significant heterogeneity (I^2^ = 17 %, *p* = 0.3) (see Additional file 3). Hospital-LOS was reported in the study by Rivers and the ProCESS, ARISE and ProMISe studies [6, 7, 30, 31]. The pooled results showed there was also no effect of EGDT on hospital-LOS (MD −0.07, 95%CI −1.12 - 0.97) with no heterogeneity (I^2^ = 0 %, *p* = 0.73). (see Additional file 4). After removing the study by Rivers [7], the pooled results was −0.11 (95%CI −1.21 - 1.00), with no heterogeneity (I^2^ = 0 %, *p* = 0.53).

**Table 2:** Characteristics of included studies on ED-LOS/ICU-LOS/Hospital-LOS

	ED-LOS			ICU-LOS			Hospital-LOS		
Author	EGDT	Usual care	P	EGDT	Usual care	P	EGDT	Usual care	P
Yan [45]	19.9 ± 2.2^a^	20.6 ± 1.9^a^	0.82	NA	NA	NA	NA	NA	NA
Rivers [7]	NA	NA	NA	NA	NA	NA	13.2 ± 13.8^a^	13 ± 13.7^a^	0.54
ProCESS [6]	NA	NA	NA	5.1 ± 6.3^a^	4.7 ± 5.8^a^	0.63	11.1 ± 10^a^	11.3 ± 10.9^a^	0.25
ARISE [30]	1.4 (0.5–2.7)^b^	2.0 (1.0–3.8)^b^	0.001	2.8 (1.4–5.1)^b^	2.8 (1.5–5.7)^b^	0.81	8.2 (4.9–16.7)^b^	8.5 (4.9–16.5)^b^	0.89
ProMISe [31]	1.5 (0.4–3.1) ^b^	1.3 (0.4–2.9)^b^	0.34	2.6 (1.0–5.8)^b^	2.2 (0.0–5.3)^b^	0.005	9 (4–21)^b^	9 (4–18) ^b^	0.46

*LOS* length of stay, *ED* emergency department, *ICU* intensive care unit, *NA* no available, *P* P value

^a^mean ± standard deviation; ^b^median(interquartile range)

### Secondary endpoint-2: mechanical ventilation rate, renal replacement therapy rate and ICU admission

Four studies [6, 7, 30, 31] reported data on mechanical ventilation rate, and the pooled results showed that there was no difference in mechanical ventilation rate between the two groups (OR 1.00, 95%CI 0.87 - 1.14) with moderate heterogeneity (I^2^ = 63 %, *p* = 0.04) (Table 3, Additional file 5). After removing the study by Rivers [7], the pooled OR was 1.04 (95%CI 0.91–1.20), with no significant heterogeneity (I^2^ = 37 %, *p* = 0.2).

**Table 3:** Characteristics of included studies on mechanical ventilation/renal replacement therapy/ICU admission

	Mechanical ventilation			Renal replacement therapy			ICU admission		
Author	EGDT	Usual care	P	EGDT	Usual care	P	EGDT	Usual care	P
Rivers [7]	65/130	84/133	0.02	NA	NA	NA	NA	NA	NA
ProCESS [6]	165/434	146/451	NA	12/382	11/397	NA	401/439	393/456	0.01
ARISE [30]	238/793	251/798	0.52	106/793	108/798	0.94	725/793	661/798	NA
ProMISe [31]	179/620	175/615	0.9	88/620	81/614	0.62	551/625	467/626	NA

*ICU* intensive care unit, *NA* no available, *P* P value

Three studies [6, 30, 31] reported data on renal replacement therapy rate, and the pooled results showed that there was no difference in renal replacement therapy rate between the two groups (OR 1.04, 95%CI 0.84, 1.28) with no heterogeneity (I^2^ = 0 %, *p* = 0.88) (see Additional file 6).

Additionally, EGDT was associated with more ICU admissions, and the pooled OR was 2.21 (95%CI 1.83 - 2.68), with no significant heterogeneity (*P* = 0.32; I^2^ = 13 %) (see Additional file 7).

### Secondary endpoint-3: intravenous fluids volume (ml), vasopressor use rate, dobutamine use rate and blood transfusion rate from 0 to 72 h

The intravenous fluids volume was reported in five studies [6, 7, 30, 31, 45] (Table 4). During the 6-h intervention period, patients in the EGDT group received a larger mean volume of intravenous fluids than did those in the control group (Table 3). Between 6 and 72 h, the pooled results of the ProCESS, ARISE and ProMISe studies [6, 30, 31] showed that there was no significant difference in intravenous fluids volume between the two groups. Between 0 and 72 h, it was seemed that patients in the EGDT group received more intravenous fluids than those in the usual care group. However, the pooled results of the ProCESS and ProMISe studies [6, 31] showed that there was no statistical significance between the two groups (see Additional file 8).

**Table 4:** Resuscitation and processes of care from baseline to 72 h

		0–6 h			6–72 h			0–72 h		
Author		EGDT	Usual care	P	EGDT	Usual care	P	EGDT	Usual care	*P*
Wang [45]	Intravenous fluids (ml)	4895 ± 210^a^	2340 ± 95^a^	0.01	NA	NA	NA	NA	NA	NA
Rivers [7]	Intravenous fluids (ml)	4981 ± 2984^a^	3499 ± 2438^a^	0.001	8625 ± 5162^a^	10602 ± 6216^a^	0.01	13443 ± 6390^a^	13358 ± 7729^a^	0.73
	Vasopressor use	32/117	36/119	0.62	34/117	51/119	0.03	43/117	61/119	0.02
	Dobutamine use	16/117	1/119	0.001	17/117	10/119	0.14	18/117	11/119	0.15
	Blood transfusion	75/117	22/119	0.001	13/117	39/119	0.001	80/117	53/119	0.001
ProCESS [6]	Intravenous fluids (ml)	2805 ± 1957^a^	2279 ± 1881^a^	0.0001	4458 ± 3878^a^	4354 ± 3882^a^	0.08	7253 ± 4605^a^	6633 ± 4560^a^	0.0001
	Vasopressor use	241/439	201/456	0.003	209/439	197/456	0.38	265/439	245/456	0.05
	Dobutamine use	35/439	4/456	0.0001	19/439	10/456	0.08	41/439	13/456	0.0001
	Blood transfusion	63/439	34/456	0.001	87/439	82/456	0.54	120/439	102/456	0.22
ARISE [30]	Intravenous fluids (ml)	1964 ± 1415^a^	1713 ± 1401^a^	0.001	4274 ± 3071^a^	4382 ± 3136^a^	0.51	NA	NA	NA
	Vasopressor use	528/793	461/798	0.001	460/782	401/778	0.004	NA	NA	NA
	Dobutamine use	122/793	21/798	0.001	74/782	39/788	0.001	NA	NA	NA
	Blood transfusion	108/793	56/798	0.001	86/782	92/778	0.61	NA	NA	NA
ProMISe [31]	Intravenous fluids (ml)	2226 ± 1443^a^	2022 ± 1271	NA	4215 ± 3068^a^	4366 ± 3114	NA	5946 ± 3740^a^	5844 ± 3651^a^	NA
	Vasopressor use	332/623	291/625	NA	349/603	317/603	NA	377/623	344/625	NA
	Dobut mine use	113/623	24/625	NA	107/603	39/603	NA	139/623	44/625	NA
	Blood transfusion	55/623	24/625	NA	76/603	51/603	NA	107/623	65/625	NA

*NA* no available, *P* P value

^a^mean ± standard deviation

The results of vasopressor use rates were showed in Table 4. The pooled results showed that more patients in the EGDT group received vasopressor in the first 6 h (OR 1.38, 95%CI 1.19–1.60), with no significant heterogeneity (I^2^ = 20 %, *p* = 0.29); From 6 to 72 h and 0 to 72 h, the pooled results showed that there was no significant difference in vasopressor use rate between the two groups, however, with significant heterogeneity (I^2^ = 68 and I^2^ = 78 %, separately). After removing the study by Rivers [7], the pooled results showed that EGDT was associated with a higher vasopressor use rate (0–6 h: OR 1.42, 95%CI 1.25–1.62; 6–72 h: OR 1.27, 95 % 1.12–1.45; 0–72 h: OR 1.28 95%CI 1.08–1.52), with no heterogeneity (I^2^ = 0 %, *p* = 0.62; I^2^ = 0 %, *p* = 0.76; I^2^ = 0 %, *p* = 0.79, separately) (see Additional file 9).

The results of dobutamine usage rate were also showed in Table 4. The pooled results showed that EGDT was associated with a higher dobutamine usage rate (0–6 h: OR 6.68, 95%CI 4.91 - 9.10; 6–72 h: OR 2.41 95%CI 1.88–3.09; 0–72 h: OR 3.40 95%CI 2.54–4.54), with no significant heterogeneity (I^2^ = 0 %, *p* = 0.55; I^2^ = 3 %, *p* = 0.38; I^2^ = 30 %, *p* = 0.24, separately). After removing the study by Rivers [7], the pooled results did not change a lot (0–6 h: OR 6.43, 95%CI 4.70 - 8.80; 6–72 h: OR 2.47 95%CI 1.91 - 3.21; 0–72 h: OR 3.72 95%CI 2.72 - 5.09), with no significant heterogeneity (I^2^ = 0 %, *p* = 0.59; I^2^ = 26 %, *p* = 0.26; I^2^ = 0 %, *p* = 0.84, separately) (see Additional file 10).

Table 4 showed the results of transfusion rate. The pooled results showed that patients in the EGDT group received more blood transfusion than those in the control group in the first 6 h and between 0 and 72 h (OR 2.91, 95%CI 1.72 - 4.91; OR 1.75, 95%CI 1.21–2.54, separately), with significant heterogeneity (I^2^ = 81 %, *p* = 0.001; I^2^ = 65 %, *p* = 0.06). However, the above effect of EGDT was not found between 6 and 72 h (OR 0.87, 95%CI 0.51–1.46) with significant heterogeneity (I^2^ = 86 %, *p* = 0.0001). After removing the study by Rivers [7], the pooled results did not change a lot (0–6 h: OR 2.16, 95%CI 1.71 - 2.73; 6–72 h: OR 1.16 95%CI 0.86 - 1.55; 0–72 h: OR 1.52 95%CI 1.12 - 2.06), with no significant heterogeneity (I^2^ = 0 %, *p* = 0.87; I^2^ = 56 %, *p* = 0.11; I^2^ = 47 %, *p* = 0.17, separately) (see Additional file 11).

### Secondary endpoint-4: adverse events

The incidences of adverse events were reported in 3 studies [6, 30, 31]. The pooled results showed that there were no effects of EGDT on the incidence of adverse events (OR 1.06, 95%CI 0.80 - 1.39) with moderate heterogeneity (I^2^ = 62 %, *p* = 0.07) (see Additional file 12).

## Discussion

Six studies were identified from 2001 to 2015 enrolling a total of 4336 patients [6, 7, 30, 31, 45, 46]. The present meta-analysis showed that there was no difference in mortality rate of patients between the EGDT group and the usual care group. Furthermore, the results of TSA indicated that the present conclusion was robust, and no further studies were required.

Although our results showed that the application of EGDT could not reduce the mortality rate of patients with severe sepsis and septic shock, one of the most plausible explanations for the decline in mortality trends in recent years was the practice of EGDT [47, 48]. The following reasons may explain these inconsistencies. Due to the appealing results of the study by Rivers, EGDT gets the recommendation of the SSC guideline, and has been regarded as the standard of care in patients with severe sepsis and septic shock in many hospitals in developed areas [7]. As in the ARISE study [30], There was no significant difference in the mean intravenous fluid volume that had been infused prior to randomization, 61.9 % of patients in the usual-care group received central venous catheter insertion and the time of initial use of intravenous antimicrobial was similar between the two groups. Similar situation was also present in the ProCESS study [6] and ProMISe study [31]. That is, the essence of EGDT (early identification and diagnosis of sepsis, early fluid resuscitation and early infection control) has been penetrated into the standard of care in patients with severe sepsis and septic shock, that is why the mortality rates of patients in the ProCESS, ARISE and ProMISe [6, 30, 31] studies were significantly lower than those in the study by Rivers [7]. Here, it should be noted that the newest 3 RCTs were conducted in developed areas (one in America [6], one in Australia [30] and one in the UK [31]). In the study by Zhu [49], the authors examined the compliance of the SSC resuscitation bundle in patients presenting to the emergency department with severe sepsis and septic shock in a university affiliated hospital in China. The results showed all elements of the bundle were completed in only 1.04 % of patients, and this number was significantly lower than that in the developed areas [50]. Another study also pointed out that the rate of compliance with resuscitation bundle in Asia was significantly lower than that in the Europe and America [51, 52]. In general, factors hindering clinicians from adhering to clinical practice guidelines include knowledge, attitude and behavior. Firstly, the imperfect understanding of the definition of severe sepsis and septic shock limits the early diagnosis of these serious conditions [49]. Secondly, insufficient training of sepsis resuscitation bundle further limits the practice of the EGDT [50, 52]. Thirdly, as the doctor-patient contradiction is aggravating gradually especially in China, more and more severe sepsis patients are hospitalized directly, instead of receiving standard resuscitation following the SSC guideline in the emergency department [53]. Fourthly, the practice of EGDT is resource-intensive. Given the insufficient number of medical staffs and disproportionate emergency visits, the application of EGDT would significantly prolong the stay in the emergency department. Meanwhile, other patients could not receive timely and effective treatment [53, 54]. Finally, in some low and middle income nations, blood culture, central venous pressure or blood gases measurement, lactate and central or mixed venous oxygen saturation measurement could not be performed directly in emergency department even in intensive care units [49, 52]. Therefore, the results of the 3 newest RCTs [6, 30, 31] do not indicate that there is no effect of EGDT on mortality rate in patients with severe sepsis and septic shock. Instead, their findings suggest the EGDT has been regarded as the standard of care in these patients particularly in developed areas. And further studies evaluating EGDT versus usual care in patients with severe sepsis and septic shock in low or moderate countries may be required.

In recent year, several meta-analysis reported that EGDT was associated with lower risk of mortality in patients with severe sepsis and septic shock [32, 55–57]. However, the rates of compliance with the SSC guideline were low in these meta-analysis, indicating that the drop in mortality is partially due to other factors. Additionally, EGDT is more capable in correcting cryptogenic shock by continuous monitoring of ScvO_2_. However, its effectiveness has been a topic of much debate and many other indicators which are more cost-effective has been proposed, including lactate concentration, base deficit, and pH [58]. In a recent study, the authors concluded that fluid resuscitation therapy under the guidance of lactate concentration rate was feasible and reliable in patients with severe sepsis and septic shock [59]. Furthermore, it has been reported that early lactate-guided therapy was associated with lower mortality rate in patients with severe sepsis and septic shock [60]. In addition, the effectiveness of CVP was also questioned in a retrospective study [61]. Excessive medical treatment is associated with unfavorable outcomes [62]. Therefore, the concept of “one size fits all” is not appropriate for a complex disease state like severe sepsis or septic shock, and the new concept of “individualized goal-directed hemodynamic therapy” has been proposed [63].

### Strengths and limitations

To our best knowledge, the present meta-analysis was the first to systematically evaluating the standard EGDT versus usual care in patients with severe sepsis and septic shock with TSA. Three multicenter RCTs (ProCESS, ARISE and ProMISe) were included [6, 30, 31]. Our search strategy was broad and irrespective of language. The study selection, data extraction and methodological evaluation were rigorously performed by two investigators independently. Another advantage of our meta-analysis was TSA. The last but not least was that we prospectively registered our study protocol with PROSPERO (International prospective register of systematic reviews; CRD42015017667).

However, several limitations of our meta-analysis should be mentioned. Firstly, some studies evaluating modified EGDT were not included. Secondly, the organizational structures of EDs or ICUs, demographic characteristics of sepsis patients, and the definitions of usual cares may be different in different areas. Thirdly, SOFA-score which was a secondary endpoint in our registered protocol was replaced by length of stay in emergency department, mechanical ventilation rate, renal replacement therapy rate and the incidence of adverse events in the present meta-analysis. Finally, the number of included studies was too small to perform publication bias examination; therefore our results may be influenced by the publication bias.

## Conclusions

The current evidence does not support the significant advantage of early goal-directed therapy (EGDT) in the resuscitation of patients with severe sepsis and septic shock. Despite the conclusion of the present meta-analysis was robust in developed areas, more designed rigorously studies are required to determine the effects of EGDT on patients with severe sepsis and septic shock in low and moderate income areas.

## Additional files

Additional file 1: Forest plot showing the effects of early goal-direced therapy on ICU, hospital, 28-day, 60-day and 90-day mortality. (DOC 20 kb) Additional file 2: Forest plot showing the effects of early goal-direced therapy on length of ICU stay. (TIF 1379 kb) Additional file 3: Forest plot showing the effects of early goal-direced therapy on length of hospital stay. (TIF 352 kb) Additional file 4: Forest plot showing the effects of early goal-direced therapy on mechnical ventilation rate. (TIF 209 kb) Additional file 5: Forest plot showing the effects of early goal-direced therapy on renal replacement therapy rate. (TIF 408 kb) Additional file 6: Forest plot showing the effects of early goal-direced therapy on ICU admission rate. (TIF 367 kb) Additional file 7: Forest plot showing the effects of early goal-direced therapy on intraveous fluids volumes. (TIF 375 kb) Additional file 8: Forest plot showing the effects of early goal-direced therapy on vasopressor use rate. (TIF 968 kb) Additional file 9: Forest plot showing the effects of early goal-direced therapy on dobutamine usage rate. (TIF 942 kb) Additional file 10: Forest plot showing the effects of early goal-direced therapy on blood transfusion rate. (TIF 946 kb) Additional file 11: Forest plot showing the effects of early goal-direced therapy on adverse events. (TIF 939 kb) Additional file 12: Search strategy (Pubmed). (TIF 369 kb)

## Abbreviations

CI: confidence interval
EGDT: early goal-directed therapy
ICU: intensive care unit
LOS: length of stay
MV: mechanical ventilation
OR: odd ratio
RCT: randomised controlled trial
ScvO2: central venous oxygen saturation
TSA: trial sequential analysis
WMD: weighted mean difference

## References

[1] Jawad I, Lukšić I, Rafnsson SB (2012). Assessing available information on the burden of sepsis: global estimates of incidence, prevalence and mortality. J Glob Health.

[2] Angus DC, Linde-Zwirble WT, Lidicker J, Clermont G, Carcillo J, Pinsky MR (2001). Epidemiology of severe sepsis in the United States: analysis of incidence, outcome, and associated costs of care. Crit Care Med.

[3] Gaieski DF, Edwards JM, Kallan MJ, Carr BG (2013). Benchmarking the incidence and mortality of severe sepsis in the United States. Crit Care Med.

[4] The Australasian Resuscitation in Sepsis Evaluation (ARISE) Investigators, Australian and New Zealand Intensive Care Society (ANZICS) Adult Patient Database (APD) Management Committee (2007). The outcome of sepsis and septic shock presenting to the emergency departments of Australia and New Zealand. Crit Care Resusc.

[5] Quenot JP, Binquet C, Kara F, Martinet O, Ganster F, Navellou JC (2013). The epidemiology of septic shock in French intensive care units: the prospective multicenter cohort EPISS study. Crit Care.

[6] Yealy DM, Kellum JA, Huang DT, Barnato AE, Weissfeld LA, Pike F (2014). A randomized trial of protocol-based care for early septic shock. N Engl J Med.

[7] Rivers E, Nguyen B, Havstad S, Ressler J, Muzzin A, Knoblich B (2001). Early goal-directed therapy in the treatment of severe sepsis and septic shock. N Engl J Med.

[8] Dellinger RP, Levy MM, Rhodes A, Annane D, Gerlach H, Opal SM (2013). Surviving Sepsis Campaign: international guidelines for management of severe sepsis and septic shock, 2012. Intensive Care Med.

[9] Dellinger RP, Levy MM, Carlet JM, Bion J, Parker MM, Jaeschke R (2008). Surviving Sepsis Campaign: international guidelines for management of severe sepsis and septic shock: 2008. Crit Care Med.

[10] Dellinger RP, Carlet JM, Masur H, Gerlach H, Calandra T, Cohen J (2004). Surviving Sepsis Campaign guidelines for management of severe sepsis and septic shock. Crit Care Med.

[11] Micek ST, Roubinian N, Heuring T, Bode M, Williams J, Harrison C (2006). Before-after study of a standardized hospital order set for the management of septic shock. Crit Care Med.

[12] Nguyen HB, Corbett SW, Steele R, Banta J, Clark RT, Hayes SR, et al. Implementation of a bundle of quality indicators for the early management of severe sepsis and septic shock is associated with decreased mortality. Crit Care Med. 2007;35:1105–12. 10.1097/01.CCM.0000259463.33848.3D 17334251

[13] Trzeciak S, Dellinger RP, Abate NL, Cowan RM, Stauss M, Kilgannon JH (2006). Translating research to clinical practice: a 1-year experience with implementing early goal-directed therapy for septic shock in the emergency department. Chest.

[14] Shapiro NI, Howell MD, Talmor D, Lahey D, Ngo L, Buras J, et al. Implementation and outcomes of the Multiple Urgent Sepsis Therapies (MUST) protocol. Crit Care Med. 2006;34:1025–32. 10.1097/01.CCM.0000206104.18647.A8 16484890

[15] Kortgen A, Niederprum P, Bauer M (2006). Implementation of an evidence-based “standard operating procedure” and outcome in septic shock. Crit Care Med.

[16] El Solh AA, Akinnusi ME, Alsawalha LN, Pineda LA (2008). Outcome of septic shock in older adults after implementation of the sepsis “bundle”. J Am Geriatr Soc.

[17] Sebat F, Johnson D, Musthafa AA, Watnik M, Moore S, Henry K, et al. A multidisciplinary community hospital program for early and rapid resuscitation of shock in nontrauma patients. Chest. 2005;127:1729–43. 10.1378/chest.127.5.1729 15888853

[18] Jones AE, Focht A, Horton JM, Kline JA (2007). Prospective external validation of the clinical effectiveness of an emergency department-based early goal-directed therapy protocol for severe sepsis and septic shock. Chest.

[19] Girardis M, Rinaldi L, Donno L, Marietta M, Codeluppi M, Marchegiano P, et al. Sopravvivere alla Sepsi Group of the Modena-University Hospital: Effects on management and outcome of severe sepsis and septic shock patients admitted to the intensive care unit after implementation of a sepsis program: a pilot study. Crit Care. 2009;13:R143. 10.1186/cc8029 PMC2784353 19728879

[20] Castellanos-Ortega A, Suberviola B, García-Astudillo LA, Holanda MS, Ortiz F, Llorca J, et al. Impact of the Surviving Sepsis Campaign protocols on hospital length of stay and mortality in septic shock patients: results of a three-year follow-up quasi-experimental study. Crit Care Med. 2010;38:1036–43. 10.1097/CCM.0b013e3181d455b6 20154597

[21] Levy MM, Rhodes A, Phillips GS, Townsend SR, Schorr CA, Beale R (2015). Surviving Sepsis Campaign: association between performance metrics and outcomes in a 7.5-year study. Crit Care Med.

[22] Ferrer R, Artigas A, Levy MM, Blanco J, González-Díaz G, Garnacho-Montero J, et al. Improvement in process of care and outcome after a multicenter severe sepsis educational program in Spain. JAMA. 2008;299:2294–303. 10.1001/jama.299.19.2294 18492971

[23] Shin HJ, Kang HL, Hwang SO, Kim H, Shin TY, Sang CK, et al. The efficacy of early goal-directed therapy in septic shock patients in the emergency department: severe sepsis campaign. Korean J Critic Care Med. 2010; 25(2):61-70.

[24] Pestaña D, Espinosa E, Sangüesa-Molina JR, Ramos R, Pérez-Fernández E, Duque M, et al. Compliance with a sepsis bundle and its effect on intensive care unit mortality in surgical septic shock patients. J Trauma. 2010;69:1282–7. 10.1097/TA.0b013e3181c4539f 20134352

[25] Miller 3rd RR, Dong L, Nelson NC, Brown SM, Kuttler KG, Probst DR, et al. Intermountain Healthcare Intensive Medicine Clinical Program: Multicenter implementation of a severe sepsis and septic shock treatment bundle. Am J Respir Crit Care Med. 2013;188:77–82. 10.1164/rccm.201212-2199OC PMC3735248 23631750

[26] Heppner HJ, Singler K, Kwetkat A, Popp S, Esslinger AS, Bahrmann P, et al. Do clinical guidelines improve management of sepsis in critically ill elderly patients? A before-and-after study of the implementation of a sepsis protocol. Wien Klin Wochenschr. 2012;124:692–8. 10.1007/s00508-012-0229-7 22948390

[27] Gao F, Melody T, Daniels DF, Giles S, Fox S (2005). The impact of compliance with 6-h and 24-h sepsis bundles on hospital mortality in patients with severe sepsis: a prospective observational study. Crit Care.

[28] Lefrant JY, Muller L, Raillard A, Jung B, Beaudroit L, Favier L, et al. Reduction of the severe sepsis or septic shock associated mortality by reinforcement of the recommendations bundle: a multicenter study. Ann Fr Anesth Reanim. 2010;29:621–8. 10.1016/j.annfar.2010.04.007 20634026

[29] Cardoso T, Carneiro AH, Ribeiro O, Teixeira-Pinto A, Costa-Pereira A (2010). Reducing mortality in severe sepsis with the implementation of a core 6-h bundle: results from the Portuguese community-acquired sepsis study (SACiUCI study). Crit Care.

[30] Peake SL, Delaney A, Bailey M, Bellomo R, Cameron PA, Cooper DJ (2014). Goal-directed resuscitation for patients with early septic shock. N Engl J Med.

[31] Mouncey PR, Osborn TM, Power GS, Harrison DA, Sadique MZ, Grieve RD (2015). Trial of early, goal-directed resuscitation for septic shock. N Engl J Med.

[32] Gu WJ, Wang F, Bakker J, Tang L, Liu JC (2014). The effect of goal-directed therapy on mortality in patients with sepsis - earlier is better: a meta-analysis of randomized controlled trials. Crit Care.

[33] Moher D, Liberati A, Tetzlaff J, Altman DG, The PRISMA Group (2009). Preferred reporting items for systematic reviews and meta-analyses: the PRISMA statement. BMJ.

[34] Bone RC, Balk RA, Cerra FB, Dellinger RP, Fein AM, Knaus WA, et al. American College of Chest Physicians/Society of Critical Care Medicine. Chest. 1992;101:1644–55.

[35] Levy MM, Fink MP, Marshall JC, Abraham E, Angus D, Cook D (2003). 2001 SCCM/ESICM/ACCP/ATS/SIS International Sepsis Definitions Conference. Crit Care Med.

[36] Higgins JPT, Green S (2011). Cochrane Handbook for Systematic Reviews of Interventions Version 5.1.0.

[37] Higgins JP, Altman DG, Gøtzsche PC, Jüni P, Moher D, Oxman AD, et al. The Cochrane Collaboration’s tool for assessing risk of bias in randomized trials. BMJ. 2011;343:d5928. 10.1136/bmj.d5928 PMC3196245 22008217

[38] Higgins JP, Thompson SG (2002). Quantifying heterogeneity in a meta-analysis. Stat Med.

[39] Higgins JP, Thompson SG, Deeks JJ, Altman DG (2003). Measuring inconsistency in meta-analyses. BMJ.

[40] DerSimonian R, Laird N (1986). Meta-analysis in clinical trials. Control Clin Trials.

[41] Egger M, Davey Smith G, Schneider M, Minder C (1997). Bias in meta-analysis detected by a simple, graphical test. BMJ.

[42] Brok J, Thorlund K, Gluud C, Wetterslev J (2008). Trial sequential analysis reveals insufficient information size and potentially false positive results in many meta-analyses. J Clin Epidemiol.

[43] Wetterslev J, Thorlund K, Brok J, Gluud C (2009). Estimating required information size by quantifying diversity in random-effects model meta-analyses. BMC Med Res Methodol.

[44] Jiang L, Jiang S, Zhang M, Zheng Z, Ma Y (2014). Albumin versus other fluids for fluid resuscitation in patients with sepsis: a meta-analysis. PLoS One.

[45] Wang XZ, Lü CJ, Gao FQ, Li XH, Yan WF, Ning FY (2006). Efficacy of goal-directed therapy in the treatment of septic shock. Zhongguo Wei Zhong Bing Ji Jiu Yi Xue.

[46] Early Goal-Directed Therapy Collaborative Group of Zhejiang Province (2010). The effect of early goal-directed therapy on treatment of critical patients with severe sepsis/septic shock: a multi-center, prospective, randomized, controlled study. ZhongguoWei Zhong Bing Ji Jiu Yi Xue.

[47] Lagu T, Rothberg MB, Shieh MS, Pekow PS, Steingrub JS, Lindenauer PK (2012). Hospitalizations, costs, and outcomes of severe sepsis in the United States 2003 to 2007. Crit Care Med.

[48] Muck A, Adams BD (2015). ACP journal club. Early goal-directed therapy did not reduce mortality more than usual care in early septic shock. Ann Intern Med.

[49] Zhu Y, Tao RJ, Shi W, Tong JJ, Lu YM (2011). A study of rate of compliance with sepsis bundle in patients with severe sepsis and septic shock in emergency department. Chin Crit Care Med.

[50] Nguyen HB, Lynch EL, Mou JA, Lyon K, Wittlake WA, Corbett SW (2007). The utility of a quality improvement bundle in bridging the gap between research and standard care in the management of severe sepsis and septic shock in the emergency department. Acad Emerg Med.

[51] Na S, Kuan WS, Mahadevan M, Li CH, Shrikhande P, Ray S (2012). Implementation of early goal-directed therapy and the surviving sepsis campaign resuscitation bundle in Asia. Int J Qual Health Care.

[52] Phua J, Koh Y, Du B, Tang YQ, Divatia JV, Tan CC (2011). Management of severe sepsis in patients admitted to Asian intensive care units: prospective cohort study. BMJ.

[53] Yin M, Meng XK, Zheng XY, Xiong LH, Zhou D, Zhang XH (2011). The implementation of early goal-directed therapy of patients with severe sepsis and septic shock in China: a multi-center study. The ninth army emergency medicine academic conference proceedings.

[54] Zhao L, Xiao B, Liu CL, Wang XY, Li B, Du XX (2014). Establishment and application of the number of outpatient and emergence visits and discharged patients prediction linear regression model. Chinese Medical Record.

[55] Wira CR, Dodge K, Sather J, Dziura J (2014). Meta-analysis of protocolized goal-directed hemodynamic optimization for the management of severe sepsis and septic shock in the emergency department. West J Emerg Med.

[56] Chamberlain DJ, Willis EM, Bersten AB (2011). The severe sepsis bundles as processes of care: a meta-analysis. Aust Crit Care.

[57] Barochia AV, Cui X, Vitberg D, Suffredini AF, O’Grady NP, Banks SM (2010). Bundled care for septic shock: analysis of clinical trials. Crit Care Med.

[58] Elliott DC (1998). An evaluation of the end points of resuscitation. J Am Coll Surg.

[59] Jones AE, Shapiro NI, Trzeciak S, Arnold RC, Claremont HA, Kline JA (2010). Lactate clearance vs central venous oxygen saturation as goals of early sepsis therapy: a randomized clinical trial. JAMA.

[60] Jansen TC, van Bommel J, Schoonderbeek FJ, Sleeswijk Visser SJ, van der Klooster JM, Lima AP (2010). Early lactate-guided therapy in intensive care unit patients: a multicenter, open-label, randomized controlled trial. Am J Respir Crit Care Med.

[61] Legrand M, Dupuis C, Simon C, Gayat E, Mateo J, Lukaszewicz AC (2013). Association between systemic hemodynamics and septic acute kidney injury in critically ill patients: a retrospective observational study. Crit Care.

[62] Chen C, Kollef MH (2014). Conservative fluid therapy in septic shock: an example of targeted therapeutic minimization. Crit Care.

[63] Saugel B, Trepte CJ, Heckel K, Wagner JY, Reuter DA (2015). Hemodynamic Management of Septic Shock: Is It Time for “Individualized Goal-Directed Hemodynamic Therapy” and for Specifically Targeting the Microcirculation?. Shock.

