# Supplementary material for: Early goal-directed resuscitation for patients with severe sepsis and septic shock: a meta-analysis and trial sequential analysis
Source: Scand J Trauma Resusc Emerg Med. 2016 Mar 5;24:23. doi: 10.1186/s13049-016-0214-7 (PMC4779580; doi:10.1186/s13049-016-0214-7)
Supplement: Additional file 1: — Forest plot showing the effects of early goal-direced therapy on ICU, hospital, 28-day, 60-day and 90-day mortality. (DOC 20 kb) [file 13049_2016_214_MOESM1_ESM.doc]

| **Search strategy（Pubmed）** | |
| --- | --- |
| #1 | Search (((sepsis[Title/Abstract] OR "septic shock"[Title/Abstract] OR "severe sepsis"[Title/Abstract] OR "septicemia"[Title/Abstract] OR "pyohemia"[Title/Abstract] OR "septicaemia"[Title/Abstract] OR "pyaemia"[Title/Abstract] OR "pyemia"[Title/Abstract])) AND ( "2001/01/01"[PDat] : "2015/04/05"[PDat] )) Filters: Publication date from 2001/11/01 to 2015/04/05 |
| #2 | Search ("goal directed“[Title/Abstract] OR “goal oriented”[Title/Abstract] OR “goal target”[Title/Abstract] OR “bundle”[Title/Abstract] OR “protocol”[Title/Abstract]) Filters: Publication date from 2001/11/01 to 2015/04/05 |
| #3 | Search (“cardiac index”[Title/Abstract] OR “cardiac output”[Title/Abstract] OR “oxygen delivery”[Title/Abstract] OR “cardiac volume”[Title/Abstract] OR “oxygen consumption”[Title/Abstract] OR “stroke volume”[Title/Abstract] OR “fluid therapy”[Title/Abstract] OR “fluid loading”[Title/Abstract] OR “fluid administration”[Title/Abstract]) Filters: Publication date from 2001/11/01 to 2015/04/05 |
| #2 OR #3 | Search ((((“cardiac index”[Title/Abstract] OR “cardiac output”[Title/Abstract] OR “oxygen delivery”[Title/Abstract] OR “cardiac volume”[Title/Abstract] OR “oxygen consumption”[Title/Abstract] OR “stroke volume”[Title/Abstract] OR “fluid therapy”[Title/Abstract] OR “fluid loading”[Title/Abstract] OR “fluid administration”[Title/Abstract])) AND ( "2001/11/01"[PDat] : "2015/04/05"[PDat] ))) OR ((("goal directed“[Title/Abstract] OR “goal oriented”[Title/Abstract] OR “goal target”[Title/Abstract] OR “bundle”[Title/Abstract] OR “protocol”[Title/Abstract])) AND ( "2001/11/01"[PDat] : "2015/04/05"[PDat] )) Filters: Publication date from 2001/11/01 to 2015/04/05 |
| #1 AND (#2 OR #3) | Search (((((((“cardiac index”[Title/Abstract] OR “cardiac output”[Title/Abstract] OR “oxygen delivery”[Title/Abstract] OR “cardiac volume”[Title/Abstract] OR “oxygen consumption”[Title/Abstract] OR “stroke volume”[Title/Abstract] OR “fluid therapy”[Title/Abstract] OR “fluid loading”[Title/Abstract] OR “fluid administration”[Title/Abstract])) AND ( "2001/11/01"[PDat] : "2015/04/05"[PDat] ))) OR ((("goal directed“[Title/Abstract] OR “goal oriented”[Title/Abstract] OR “goal target”[Title/Abstract] OR “bundle”[Title/Abstract] OR “protocol”[Title/Abstract])) AND ( "2001/11/01"[PDat] : "2015/04/05"[PDat] ))) AND ( "2001/11/01"[PDat] : "2015/04/05"[PDat] ))) AND (((((sepsis[Title/Abstract] OR "septic shock"[Title/Abstract] OR "severe sepsis"[Title/Abstract] OR "septicemia"[Title/Abstract] OR "pyohemia"[Title/Abstract] OR "septicaemia"[Title/Abstract] OR "pyaemia"[Title/Abstract] OR "pyemia"[Title/Abstract])) AND ( "2001/01/01"[PDat] : "2015/04/05"[PDat] ))) AND ( "2001/11/01"[PDat] : "2015/04/05"[PDat] )) Filters:Publication date from 2001/11/01 to 2015/04/05 |
