# Supplementary figures and images for: Early goal-directed resuscitation for patients with severe sepsis and septic shock: a meta-analysis and trial sequential analysis
Source: Scand J Trauma Resusc Emerg Med. 2016 Mar 5;24:23. doi: 10.1186/s13049-016-0214-7 (PMC4779580; doi:10.1186/s13049-016-0214-7)

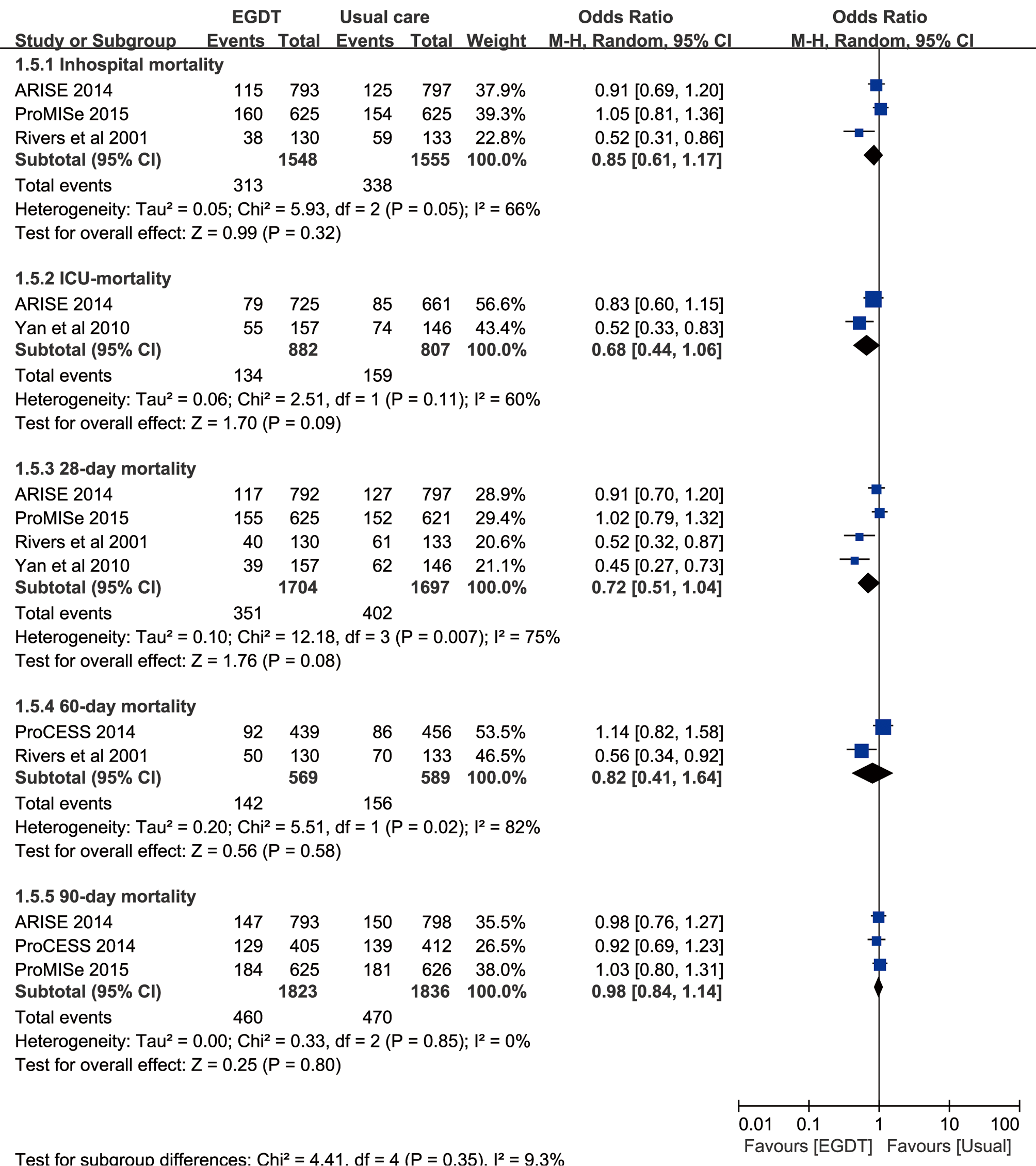

Supplement: Additional file 2: — Forest plot showing the effects of early goal-direced therapy on length of ICU stay. (TIF 1379 kb) [file 13049_2016_214_MOESM2_ESM.tif]

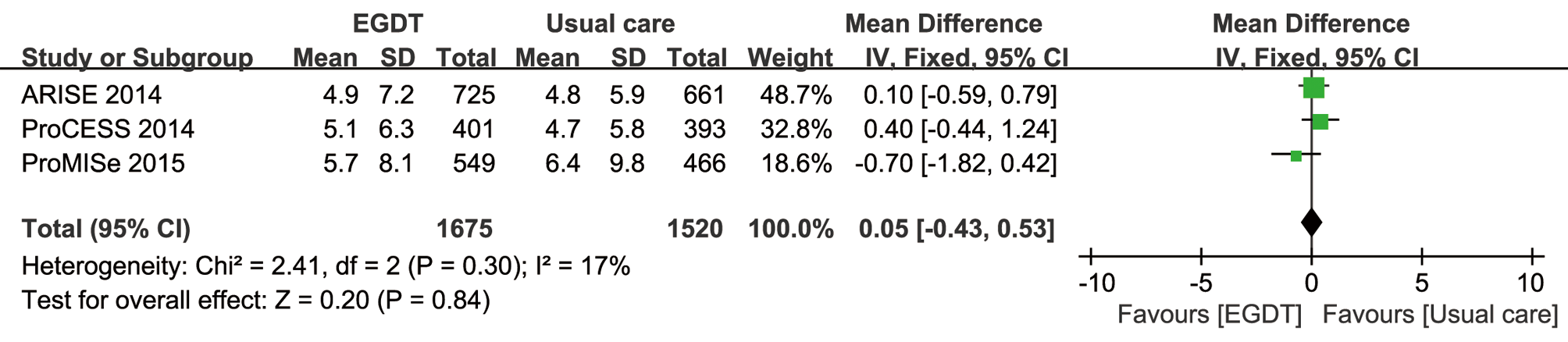

Supplement: Additional file 3: — Forest plot showing the effects of early goal-direced therapy on length of hospital stay. (TIF 352 kb) [file 13049_2016_214_MOESM3_ESM.tif]

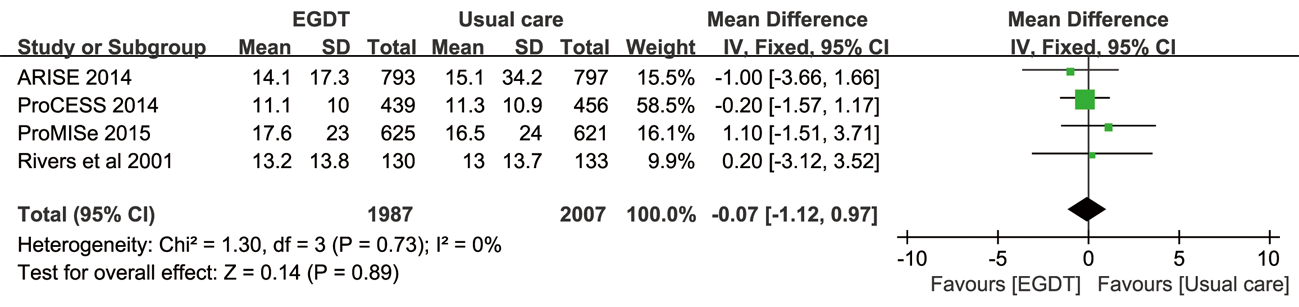

Supplement: Additional file 4: — Forest plot showing the effects of early goal-direced therapy on mechnical ventilation rate. (TIF 209 kb) [file 13049_2016_214_MOESM4_ESM.tif]

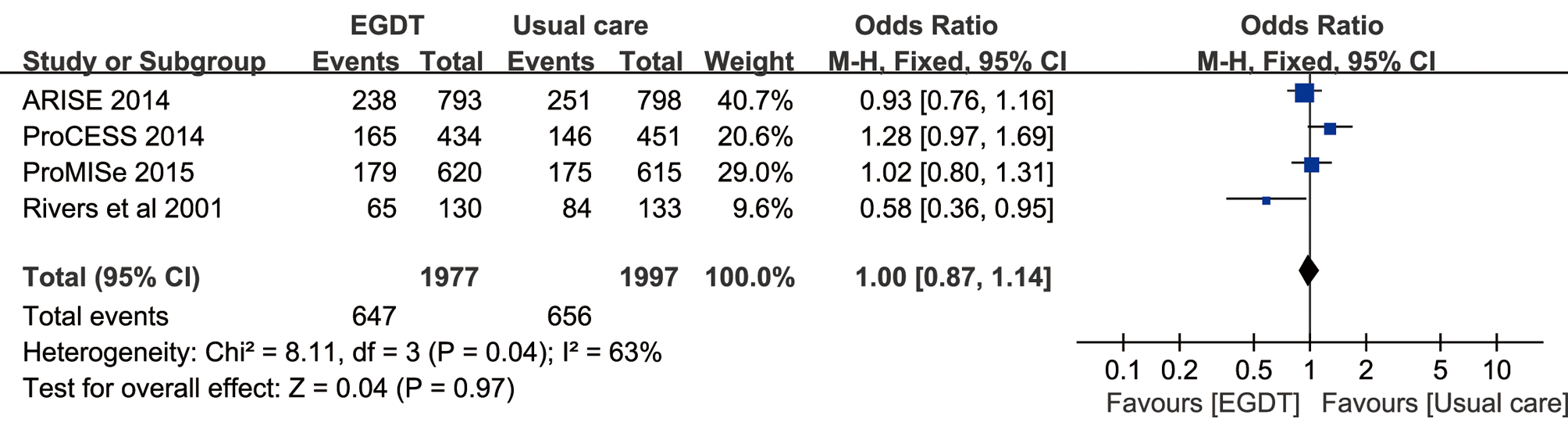

Supplement: Additional file 5: — Forest plot showing the effects of early goal-direced therapy on renal replacement therapy rate. (TIF 408 kb) [file 13049_2016_214_MOESM5_ESM.tif]

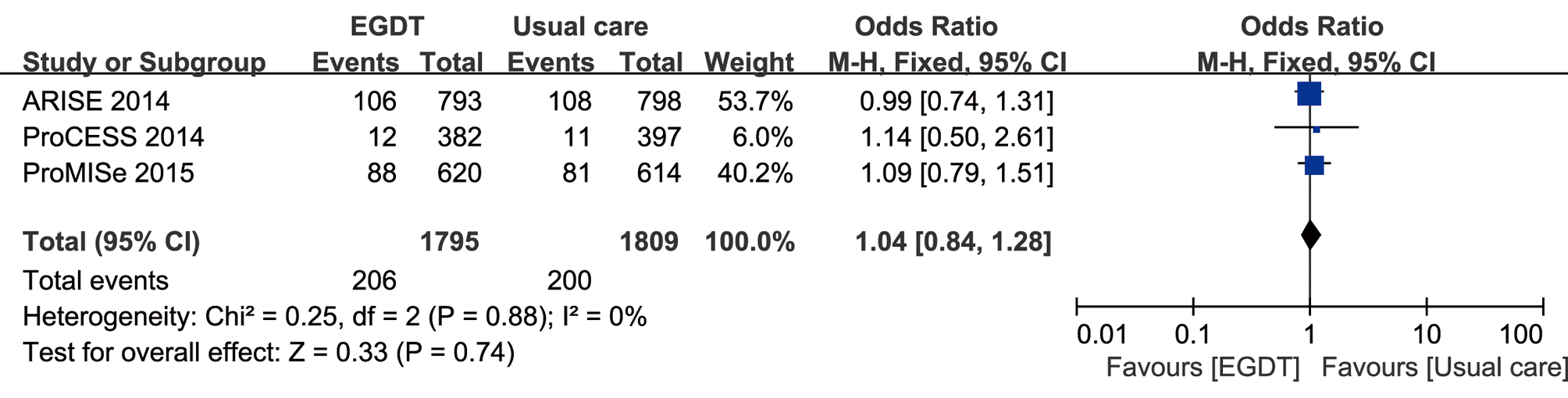

Supplement: Additional file 6: — Forest plot showing the effects of early goal-direced therapy on ICU admission rate. (TIF 367 kb) [file 13049_2016_214_MOESM6_ESM.tif]

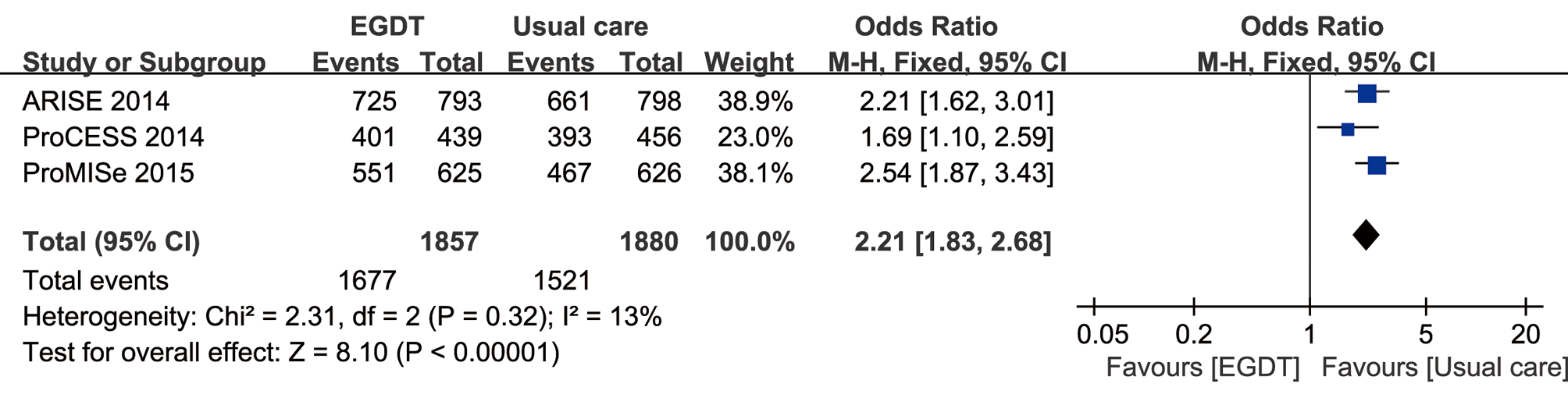

Supplement: Additional file 7: — Forest plot showing the effects of early goal-direced therapy on intraveous fluids volumes. (TIF 375 kb) [file 13049_2016_214_MOESM7_ESM.tif]

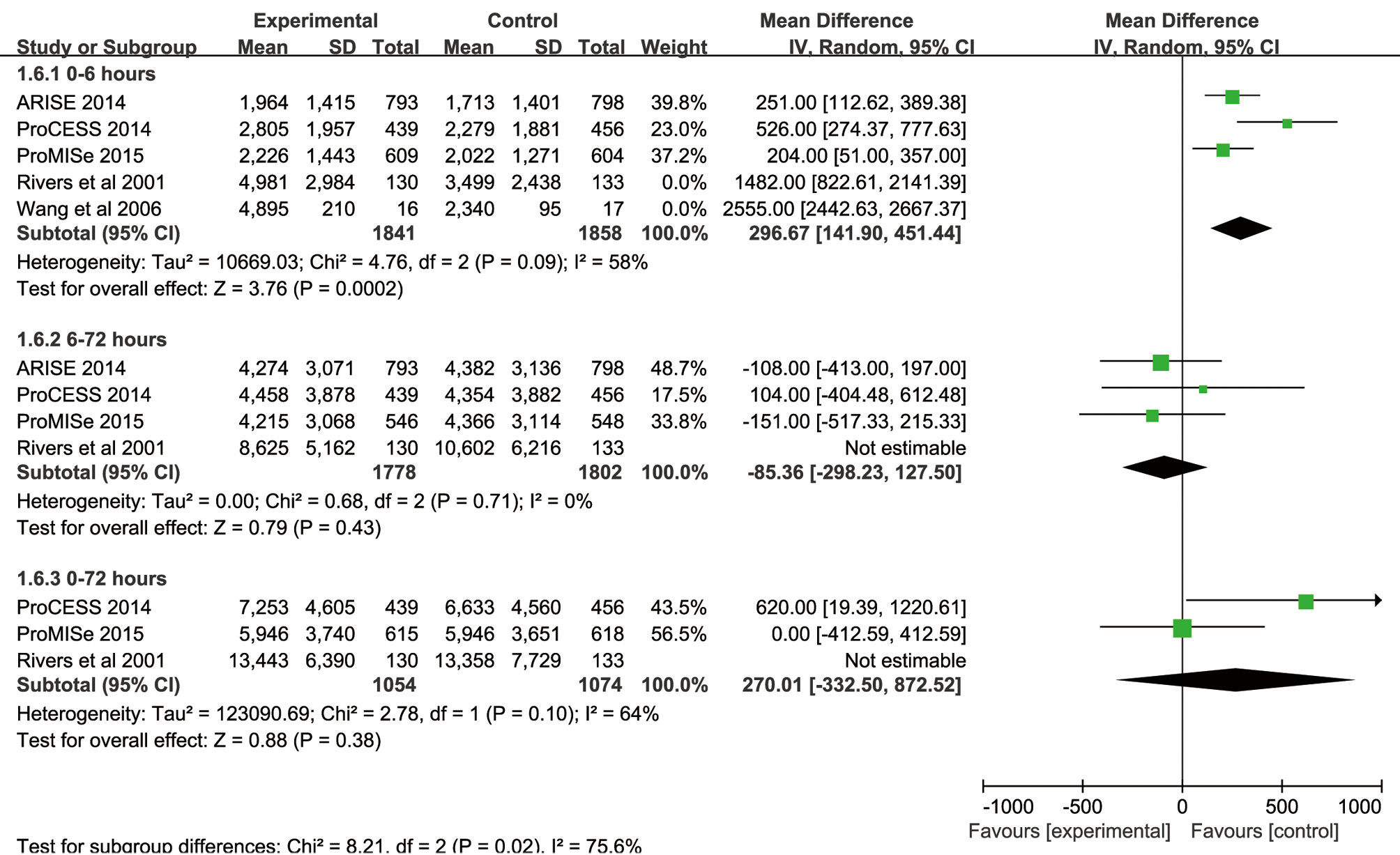

Supplement: Additional file 8: — Forest plot showing the effects of early goal-direced therapy on vasopressor use rate. (TIF 968 kb) [file 13049_2016_214_MOESM8_ESM.tif]

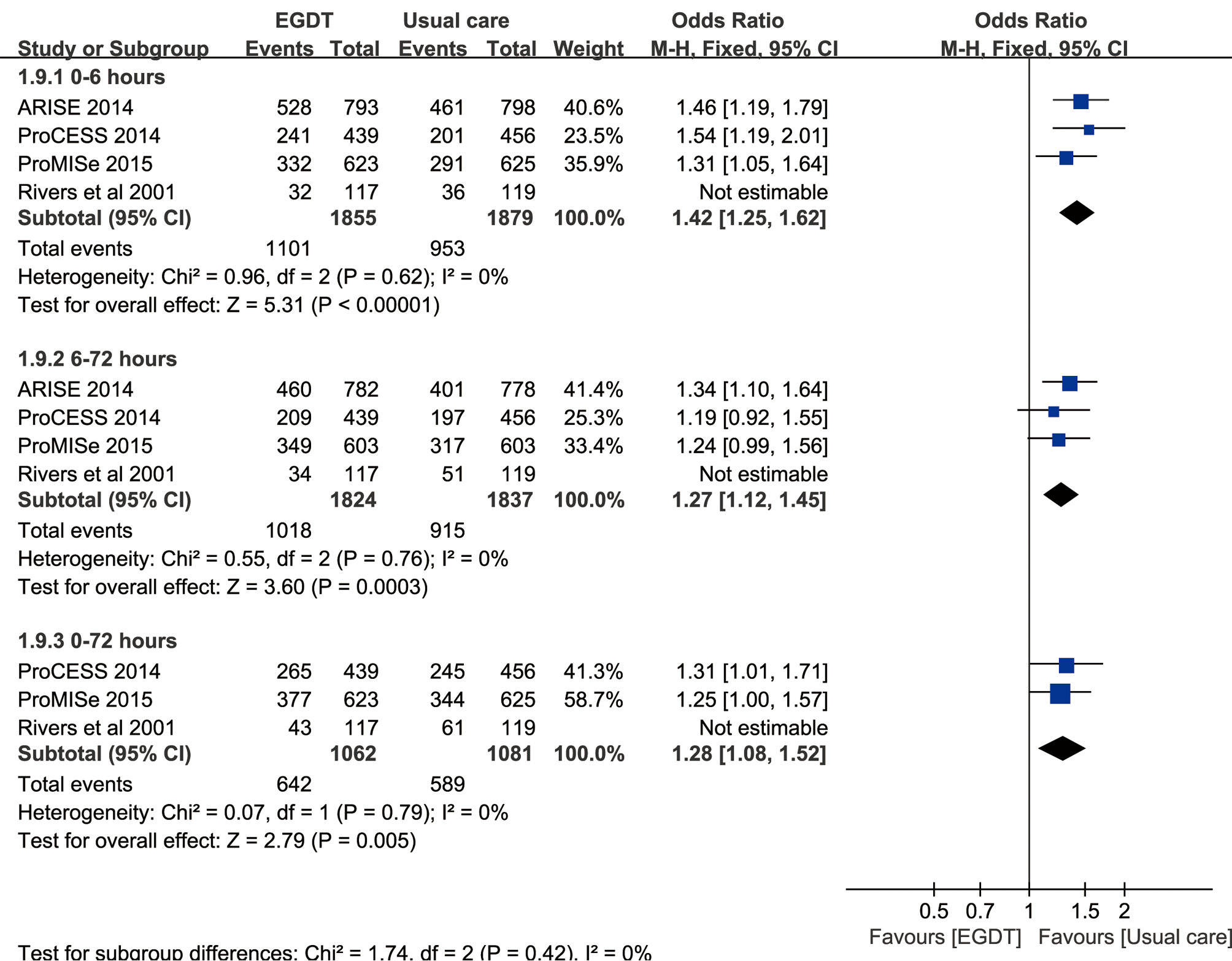

Supplement: Additional file 9: — Forest plot showing the effects of early goal-direced therapy on dobutamine usage rate. (TIF 942 kb) [file 13049_2016_214_MOESM9_ESM.tif]

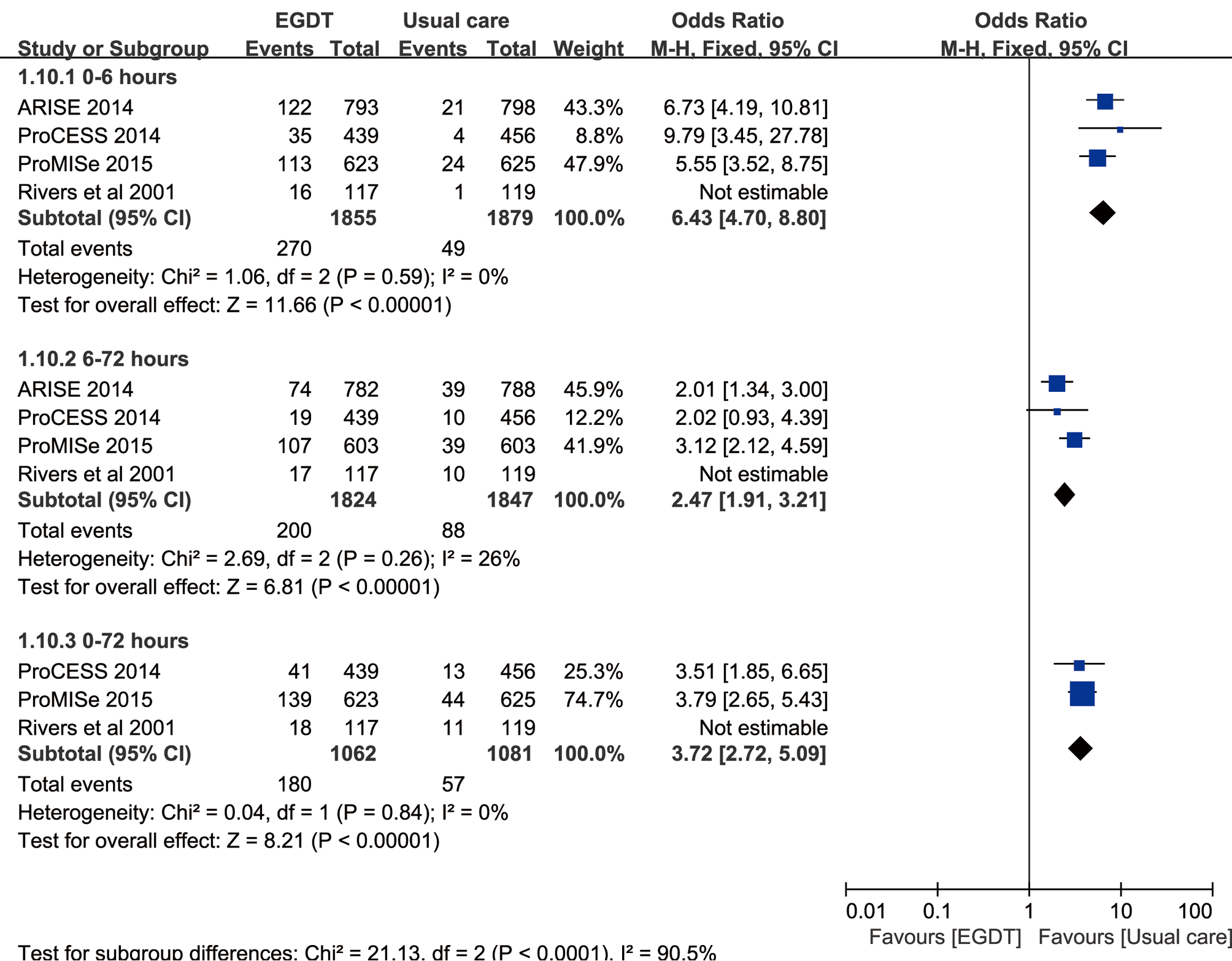

Supplement: Additional file 10: — Forest plot showing the effects of early goal-direced therapy on blood transfusion rate. (TIF 946 kb) [file 13049_2016_214_MOESM10_ESM.tif]

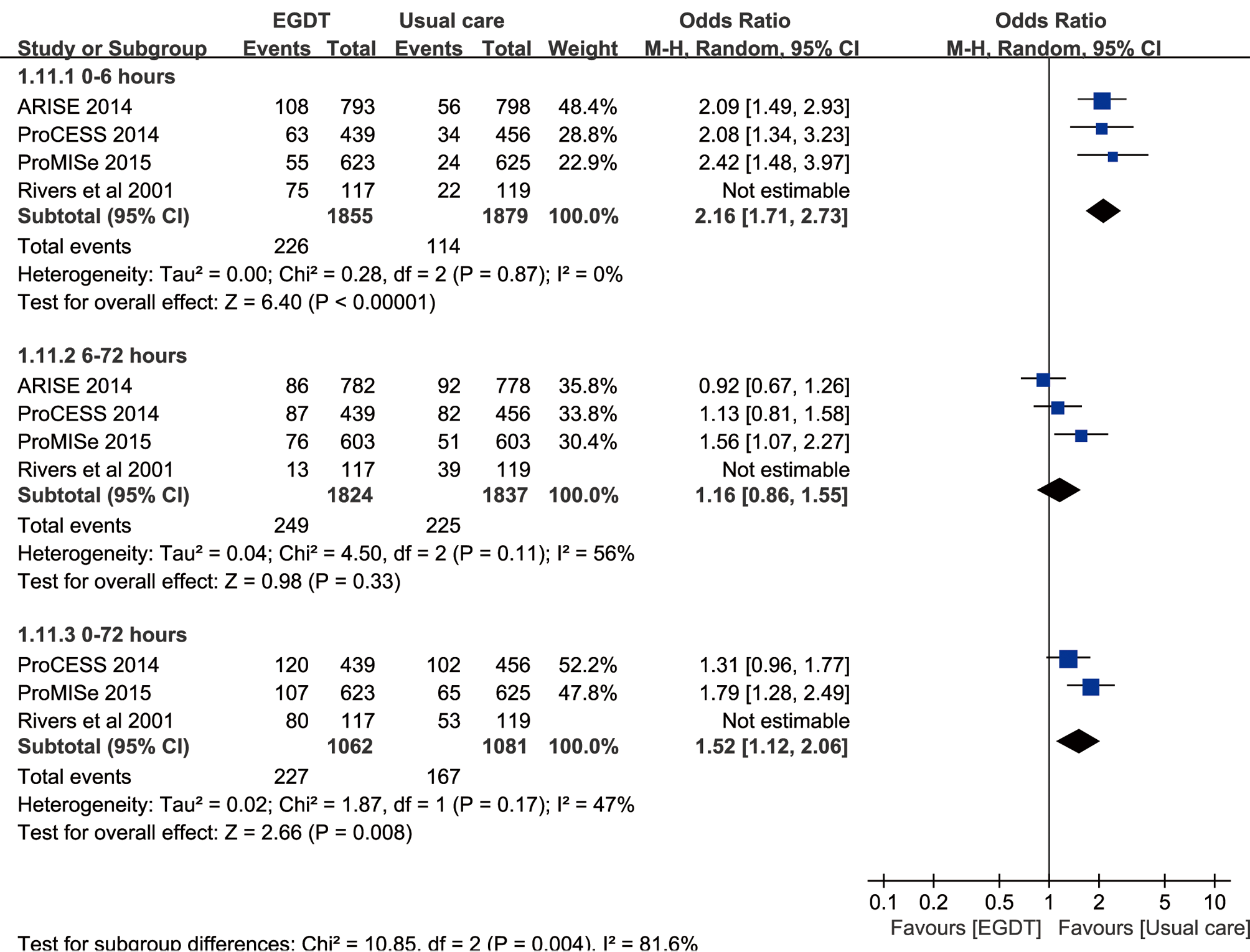

Supplement: Additional file 11: — Forest plot showing the effects of early goal-direced therapy on adverse events. (TIF 939 kb) [file 13049_2016_214_MOESM11_ESM.tif]

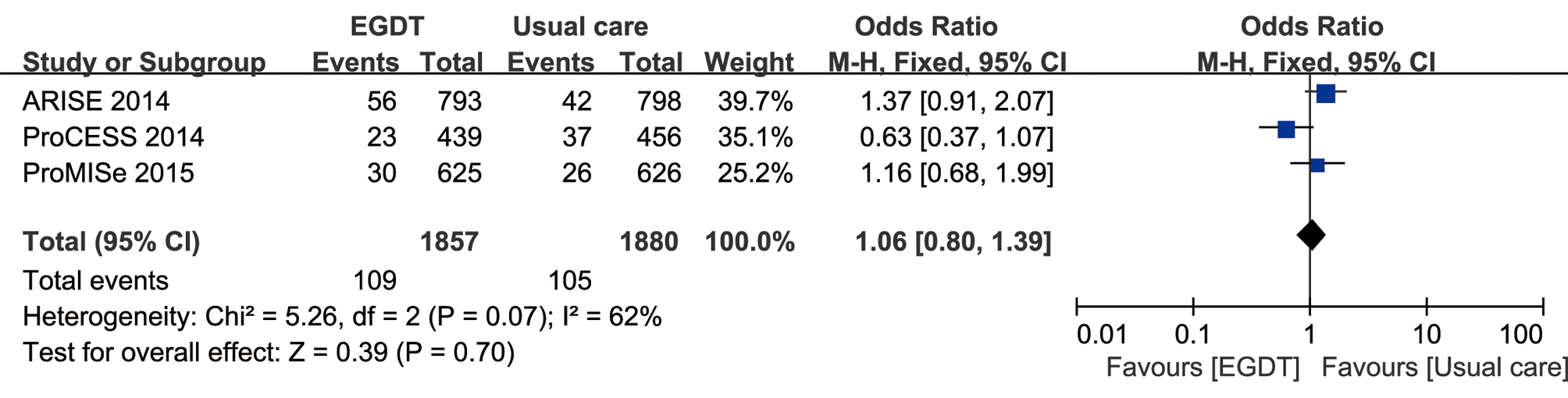

Supplement: Additional file 12: — Search strategy (Pubmed). (TIF 369 kb) [file 13049_2016_214_MOESM12_ESM.tif]
